# Diagnosis of Coronary Artery Aneurysm in a Caucasian Population Cohort: Evaluating the Agreement Between Japanese Criteria and Different Z Score Formulas

**DOI:** 10.3390/jcm14186581

**Published:** 2025-09-18

**Authors:** Belén Pastor-Villaescusa, Guido Mandilaras, Julia Weißer, Joseph Pattathu, Nikolaus A. Haas, André Jakob

**Affiliations:** 1 Department of Pediatric Cardiology, Ludwig-Maximilians-University of Munich, Marchioninistr. 15, 81377 Munich, Germany; belen.pastor@imibic.org (B.P.-V.); guidomandilaras@googlemail.com (G.M.); weisser.julia@web.de (J.W.); joseph.pattathu@med.uni-muenchen.de (J.P.); nikolaus.haas@med.uni-muenchen.de (N.A.H.); 2 Metabolism and Investigation Unit, Reina Sofia University Hospital, Maimonides Biomedical Research Institute of Cordoba (IMIBIC), University of Cordoba, 14004 Córdoba, Spain; 3 Center for Congenital Heart Defects Stuttgart, Pediatric Intensive Care, Pneumology and Allergology, Klinikum Stuttgart, Olgahospital/Women’s Clinic, 70174 Stuttgart, Germany

**Keywords:** Kawasaki disease, coronary artery aneurysm, echocardiography, Z score

## Abstract

**Background/Objectives**: Evaluating coronary artery abnormalities (CAAs) in Kawasaki disease (KD) is essential for treatment decisions and long-term management and prognosis. Accurate diagnosis is challenging due to differing criteria across guidelines. This study aimed to assess the variability in CAA prevalence using Japanese Ministry of Health (JMH) criteria and Z score formulas and identify the formula pair with the highest CAA diagnostic agreement. **Methods**: Echocardiographic data from 309 patients with acute KD were collected. CAA prevalence was evaluated using JMH criteria and Z score formulas of Kobayashi, de Zorzi, Kurotobi, McCrindle, Olivieri and Dallaire. Prevalence differences were analyzed using McNemar’s t-tests, Z score values with paired samples t-test, and agreement between Z score formula pairs with Cohen’s Kappa (κ) coefficients and Bland–Altman plots. **Results**: The CAA prevalence varied significantly across definitions. For the right CA, prevalence was lower by JMH criteria than by Z scores (32.7% vs. 37.2–39.8%). For the left main CA, JMH (47.6%) and Kobayashi (44.8%) showed higher prevalence compared to other formulas (25.8–42.9%). Variability was greater at higher Z score values (>5 mm, medium/large aneurysm). Overall, the Kobayashi–Dallaire and McCrindle–Dallaire pairs showed the highest agreement (κ = 0.745–0.831 and 0.569–0.870, respectively); however, the McCrindle–Dallaire reached only moderate agreement for the left main CA (κ = 0.569). **Conclusions**: The Kobayashi and Dallaire formulas appear most suitable for evaluating CAA in predominantly Caucasian populations. Larger validation studies are warranted to refine diagnostic criteria and optimize global KD care.

## 1. Introduction

Kawasaki disease (KD) is a pediatric systemic vasculitis of unknown etiology, primarily affecting infants and young children [1,2]. In Germany, the KD incidence is estimated to be 7.2 per 100,000 in children under 5 years of age [3].

In untreated patients, approximately 25% develop coronary artery abnormalities (CAAs), including dilation and aneurysm formation, which are major contributors to long-term cardiovascular events and increased mortality [4]. Accurate evaluation of CAAs is essential for guiding early treatment intensification, plays a central role in the diagnosis of incomplete KD, and ultimately influences long-term prognosis [1,5]. However, CAA characterization varies according to different guidelines and criteria [1,5], posing challenges for physicians in making accurate diagnoses of KD patients.

The classification of CAAs by the Japanese Ministry of Health (JMH), as described in the 2020 guidelines by Fukazawa et al. [6], employs both absolute measurements and Z scores to ensure age-appropriate evaluation across different pediatric age groups. For children under five, the classification primarily relies on absolute internal lumen diameters: small aneurysms measure less than 4 mm, medium aneurysms are between 4 mm and 8 mm, and large aneurysms exceed 8 mm. This clear-cut approach in younger children is designed to accommodate the relative consistency in CA size during early childhood, simplifying the detection of significant coronary changes.

In children aged five years and older, the guidelines incorporate Z scores to account for individual body size and growth variations. For this older age group, a small aneurysm is defined by a Z score of 2.5 to less than 5, a medium aneurysm has a Z score between 5 and less than 10 or an absolute dimension under 8 mm, and a large aneurysm is indicated by a Z score of 10 or higher or an absolute diameter of 8 mm or more. Using Z scores in this age category allows a more accurate assessment of CA enlargement relative to normative data, thereby reducing the risk of over- or underestimating aneurysm severity based on body surface area (BSA) adjustments.

While the JMH criteria are commonly employed due to their simplicity and quick CAA assessment, it does not account on CA dimensions depending on body size in children with less than five years of age. As a result, these criteria may not consistently detect dilated CAs and underestimate the true prevalence of CAAs, particularly in young infants [7].

The 2004 American Heart Association (AHA) guidelines, updated in 2017 and 2024, [8] proposed criteria based solely on CA Z scores. These scores, expressed as standard deviation (SD) units from the mean and adjusted for BSA, define CA dilatation as a Z score ≥ 2.0 and CA aneurysm as a Z score ≥ 2.5 [1,9]. It is important to note that a Z score, particularly when indicating CA dilation, neither confirms nor excludes arterial wall damage, whereas the presence of an anatomic aneurysm does. Thus, Z scores alone cannot determine the nature of CA injury. Nevertheless, previous studies have shown that the JMH criteria are less sensitive in detecting CAA than Z score models. For instance, de Zorzi et al. reported that 27% of KD patients classified as normal by JMH criteria had Z scores exceeding 2 [10]. Multiple CA Z score calculation methods have since been developed in different ethnic populations, leading to potential variations in respective results that could influence correct diagnosis and treatment strategies for KD patients. Although data on healthy Caucasian children are recently available [11], studies evaluating Z score models for diagnosing CAA in the European KD population remain limited. The updated KD AHA guidelines emphasize once again that a standardized Z score model has not yet been established. They recommend that, at a minimum, each center consistently should use a single Z score system over time [8].

This study aims to (1) assess the variability in CAA prevalence according to the JMH guidelines and various Z score formulas, (2) examine the discrepancies between commonly used Z score formulas, and (3) identify the pair of Z score formulas with the highest level of agreement. Ultimately, we aim to provide evidence to support recommendations for the general use of one or two specific Z score formulas, particularly for a predominantly Caucasian population.

## 2. Materials and Methods

### 2.1. Patients

For this study, echocardiographic reports of KD patients were recruited from the active, prospective population-based German Pediatric Surveillance Study for Rare Diseases (ESPED) and the Ludwig Maximilian University (LMU) Munich database between January 2011 and December 2022. All echocardiographic studies were conducted or reviewed by pediatric cardiologists trained in KD imaging. At LMU, inter-observer consistency was ensured by periodic joint reading sessions, and >60% of scans underwent dual review. All patients were pseudonymously recruited using a standardized questionnaire that collected detailed acute-phase data, including anthropometric measures and echocardiographic findings of CA diameters. Cases were re-evaluated to confirm their classification as complete or incomplete KD according to AHA criteria [3,12,13]. A subset of families provided informed consent for identity disclosure, which allowed for retrieval of complete echocardiographic records.

Out of 351 patients with echocardiographic reports, 309 were analyzed. The remaining 42 were excluded due to incomplete data, specifically missing CA measurements and/or anthropometric variables (height or weight), which are essential for Z score computation. In several of these cases, CA were qualitatively described as “normal” without numeric values. Since Z score formulas require precise quantitative input, these cases could not be included. Although this exclusion may slightly overestimate CAA prevalence, it reflects real-world documentation practices in pediatric echocardiography.

Approval of the study was provided by the Ethics Committees of the University of Freiburg and the Ludwig-Maximilians-University of Munich, in accordance with the ethical standards of the Declaration of Helsinki.

### 2.2. Coronary Artery Evaluations

Six Z score formulas (Kobayashi [14], de Zorzi [10], Kurotobi [15], McCrindle [16], Olivieri [17] and Dallaire [18]) were applied on all the available CA diameters. These models were selected based on their widespread use. Z scores were calculated for the proximal segments of the right coronary artery (RCA), left main coronary artery (LMCA), and left anterior descending artery (LAD). For the left circumflex artery (LCx), only the Kobayashi and Dallaire models provide Z score estimations.

### 2.3. Statistical Analysis

Because the data were not normally distributed, continuous variables for demographic and clinical characteristics were expressed as median and interquartile range. Categorical variables were presented as frequency counts (percentages). The statistical differences in aneurysm prevalence between the JMH criteria and the six different Z score formulas were assessed using the paired McNemar’s t-test. Differences in CA Z score values based on the six formulas for all area measurements (RCA, LMCA, LAD, and LCx) were analyzed using paired samples *t*-tests (listwise). Graphical correlations of the formulas with the CA diameters were created to visually examine the dispersion of Z scores for the RCA, LMCA, LAD, and LCx measurements.

To assess agreement in the CA Z scores across the six formulas, Cohen’s Kappa coefficients were calculated, with the following interpretation levels: poor agreement < 0; slight agreement = 0–0.20; fair agreement = 0.21–0.40; moderate agreement = 0.41–0.60; substantial agreement = 0.61–0.80; and almost perfect agreement = 0.81–1 [19]. Bland–Altman plots were created to visually examine agreement between paired Z scores across the entire range of values. Each plot shows the difference between two Z scores against their average. For ease of interpretation, we indicated the range of conventional clinical categories of CAA severity on the x-axis: no aneurysm (<2), dilation/small aneurysm (2–4.99), and medium-large aneurysm (5–20).

All statistical tests were performed using a two-sided 5% significance level. Statistical analyses were performed using SPSS software, version 24 (SPSS Inc., Chicago, IL, USA). All figures were generated using GraphPad Prism, version 6 (GraphPad Software, Boston, MA, USA).

## 3. Results

### 3.1. Study Population

The study cohort consisted of 309 patients (204 males) with available data on anthropometry and at least one CA main segment. The median age of the patients at the acute phase of KD was 32.3 months, with the youngest patient being 1 month old and the oldest 16 years of age. Details on anthropometry and dimensions of the available different coronary arteries are shown in Table 1.

### 3.2. Comparison of JMH Criteria and Z Score Formulas for CAA Prevalence Rates

Table 2 presents the general CAA prevalence rates (%) based on the JMH criteria, the six different Z score formulas, and their respective statistical differences. For the RCA, 32.7% of the patients were diagnosed with dilation or aneurysm according to the JMH criteria. This prevalence was significantly lower than the CAA rates indicated by the Z score formulas developed by Kobayashi, de Zorzi, Kurotobi, and McCrindle, which suggested higher rates ranging from 37.2% to 39.8%. In contrast, for the LMCA, the JMH criteria (47.6%) and Kobayashi formula (44.8%) showed similar and significantly higher prevalence rates than the remaining Z score formulas (25.8–42.9%). In the case of the LAD and LCx, differences in prevalence rates were less pronounced.

### 3.3. Comparison of Z Score Formulas for Caa Diagnosis

Table 3 shows each formula’s calculated Z score values across the main CA branches. Detailed *p*-values for each paired test are provided in Table S1. Mean values varied significantly between the applied Z score formulas, with most of the pair formulas showing *p*-values < 0.001, especially for the LMCA segment; however, some formulas, such as those by Kobayashi, de Zorzi, Kurotobi and Dallaire, yielded similar Z score values for the RCA (*p*-values > 0.05).

**Figure 1:**
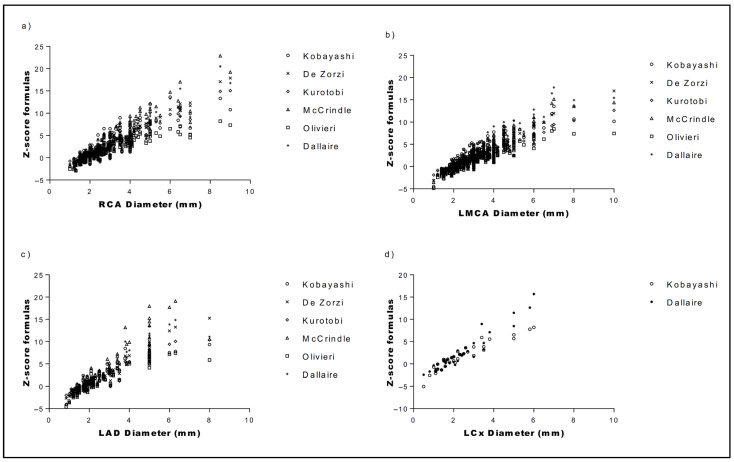
Correlation of RCA (**a**), LMCA (**b**), LAD (**c**), and LCx (**d**) diameters with all Z score formulas. LAD, left anterior descending; LCx, left circumflex; LMCA, left main coronary artery; RCA, right coronary artery.

Figure 1 illustrates the variation among the CA Z scores for RCA, LMCA, LAD, and LCx across the six Z score formulas. The variation was greater at higher Z score values, particularly for diameters > 5 mm (medium/large aneurysm). The formulas by Olivieri and Kobayashi displayed a lower trend line, reflecting lower Z score values, while McCrindle and de Zorzi exhibited higher trend lines, indicating higher Z scores.

**Figure 2:**
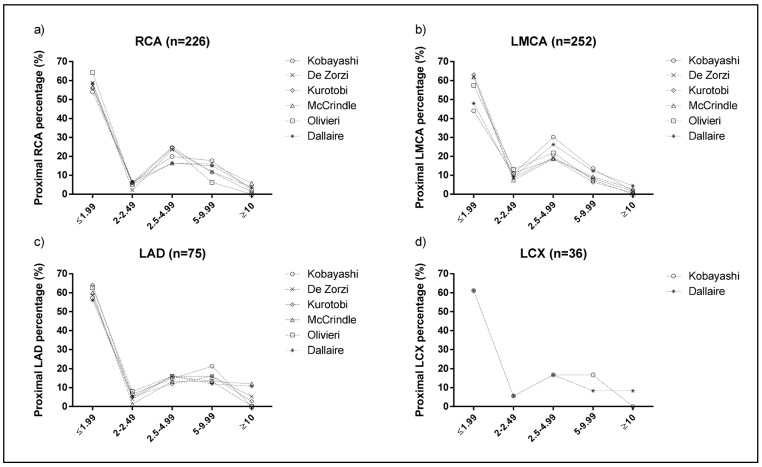
Prevalence stratification of aneurysm (RCA (**a**), LMCA (**b**), LAD (**c**), and LCx (**d**)) based on the severity (dilation: Z score 2–2.49; small aneurysm: 2.5–4.99; medium aneurysm; Z score 5–9.99; large aneurysm: Z score ≥ 10) according to Z score formulas. LAD, left anterior descending; LCx, left circumflex; LMCA, left main coronary artery; RCA, right coronary artery.

Figure 2 shows the prevalence of CAA stratified by severity. Overall, the prevalence of dilation (Z score 2–2.49) was lower across all measurements, as was the prevalence of large aneurysms (Z score ≥ 10). The highest prevalence was observed in small aneurysms (Z score 2.5–4.99).

Based on Cohen’s Kappa coefficient, Kobayashi and Dallaire showed substantial to almost perfect agreement across all dimensions, particularly for LMCA and LCx (Table 4). Similarly, de Zorzi and Kurotobi demonstrated high levels of agreement. McCrindle and Dallaire also exhibited almost perfect levels of agreement in RCA and LAD, although only a moderate agreement was seen in LMCA. The lowest level of agreement was observed between Kobayashi and Kurotobi in LMCA. Olivieri and Dallaire did not indicate a high level of agreement in any of the dimensions.

**Figure 3:**
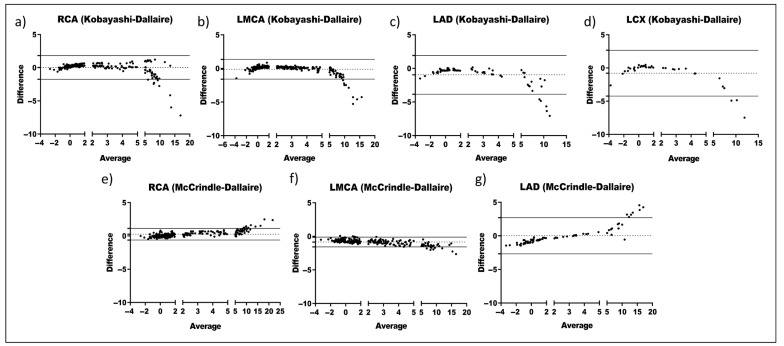
Bland–Altman plots comparing two pairs of Z scores formulas across the CA segments (RCA, right coronary artery; LMCA, left main coronary artery; LAD, left anterior descending; LCx, left circumflex): For each CA, the differences were calculated by subtracting Kobayashi’s values from Dallaire’s values (**a**–**d**), and McCrindle’s values from Dallaire’s values (**e**–**g**). The dotted line represents the average difference in Z scores, and the solid lines represent the limits of agreement for the average difference in Z scores.

**Figure 4:**
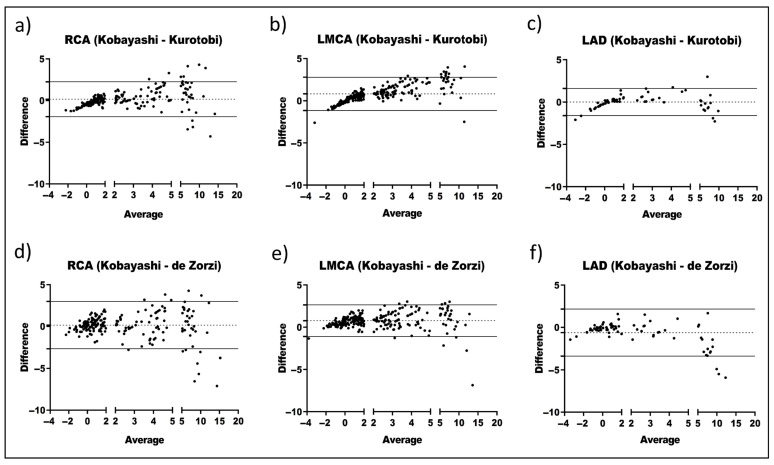
Bland–Altman plots comparing two pairs of Z scores formulas across the CA segments (RCA, right coronary artery; LMCA, left main coronary artery; LAD, left anterior descending): For each CA, the differences were calculated by subtracting Kobayashi`s values from and Kurotobi’s values (**a**–**c**) and Kobayashi’s values from de Zorzi’s values (**d**–**f**). The dotted line represents the average difference in Z scores, and the solid lines represent the limits of agreement for the average difference in Z scores.

To further evaluate the pair’s agreement and determine whether the variability of the discrepancy between two formulas is attributed to Z score values, Bland–Altman plots were assembled indicating the three ranges based on the CAA severity (no aneurysm: <2; dilation/small aneurysm: 2–4.99; medium-large aneurysm: 5–20). Kobayashi and Dallaire showed a low variability for “no aneurysm” and “dilation/small aneurysm” (Figure 3a–d). Furthermore, especially for RCA and LMCA, no wide limits of agreement were observed. McCrindle and Dallaire also displayed a low variability and narrow limits of agreement in RCA (Figure 3e–g). Nevertheless, the limits of agreement for LMCA do not include zero, indicating a considerable mean difference between the McCrindle and Dallaire formulas. Other pairs, such as Kobayashi–Kurotobi (Figure 4a–c) and Kobayashi–de Zorzi (Figure 4d,e), did not have a good agreement since the dispersion is broad, and therefore, the limits of agreements as well. On the other hand, some pairs showed a trend since the mean difference tended to be more prominent as the average increased (i.e., Kobayashi–Kurotobi in RCA and LMCA (Figure 4a,b), de Zorzi–McCrindle (Figure S1), Kurotobi and Dallaire (Figure S2), or McCrindle and Olivieri (Figure S3) both in all measurements).

Overall, the frequency of points outside the limits of agreement indicates low agreement for diagnosing medium-large aneurysms, especially pronounced in some pairs, such as Kobayashi–McCrindle in RCA and Olivieri–Dallaire in all measurements.

## 4. Discussion

An accurate assessment of CAA in KD patients is crucial for appropriate clinical management. This study assessed the variability in CAA prevalence in KD patients using the JMH criteria and six different Z score formulas frequently referenced by the AHA in a German, mainly Caucasian cohort. Our findings revealed significant differences in the prevalence of CAA between the JMH criteria and the different Z score models applied. The JMH criteria are widely applied in clinical practice, being simple and intuitive for younger age groups, and thus serve as a common reference point; however, they should not be considered a gold standard. Their limitations in detecting CA abnormalities have been highlighted, particularly because categorical determinations do not adequately reflect changes over time, whereas Z scores do. In this regard, several previous studies have advocated using Z score models as they may offer higher sensitivity in detecting CAA compared to the JMH criteria [7,10,20]. Z scores may better reflect the effect of CAA on blood flow disturbance because they are based on the relationship between the CAA size and the expected size of CA branches relative to the patient’s BSA [7]. In this context, Cantinotti et al. recently proposed new coronary Z score equations based on a large Italian pediatric population, aiming to improve accuracy across a wide BSA range [11]. Their work underscores the ongoing need for population-specific validation studies in Europe. Similarly, Lin et al. proposed reference ranges for the CA diameters in Taiwanese children younger than 6 years of age, fitting the model to correspond with BSA [21]. Nevertheless, these equations were not included in our comparative analysis as we focused on the six Z score formulas frequently referenced by the AHA guidelines.

Z scores simplify the clinical interpretation of echocardiographic results [22]. However, variations are inevitable since Z score formulas are derived from regression calculations conducted by various medical centers using average values from different populations. These differences in regression curves and parameters may lead to discrepancies in Z values, even for identical measurements. Understanding the agreement between these models is essential for enhancing diagnostic accuracy, especially given the importance of precise echocardiographic serial monitoring of CA dimensions. Such monitoring is valuable in identifying dimension increases, which may aid in diagnosing KD [23].

For RCA measurements, the prevalence rates of aneurysm using the six Z score formulas were significantly higher (37.2–39.8%) than that using the JMH criteria (32.7%), except for the Olivieri (30.5%) and Dallaire (35%) formulas, which showed comparable rates of prevalence to the JMH criteria. These findings differ from those reported by Kim et al. (2021) in a Korean population, where the Z score methods produced lower prevalence rates than the JMH criteria [24]. Additionally, authors from China have suggested that the JMH criteria underestimate the prevalence of dilation [25,26] and CAA severity, particularly in infants and younger children [7,20,24]. For LMCA aneurysms, the prevalence rates using the de Zorzi, Kurotobi, McCrindle, and Olivieri formulas (25.8–30.6%) were lower than those using the JHM criteria (47.6%), while the Dallaire (42.9%) and Kobayashi (44.8%) formulas produced slightly lower or similar rates. This suggests that the Dallaire and Kobayashi formulas are more sensitive in detecting LMCA aneurysms, which aligns with previous reports in a Chinese population [26]. However, the different types of coronary circulation must be considered, as they can lead to significant variations in lumen caliber, especially in the case of the LMCA. In this sense, prior reports have shown greater variability in LMCA measurement and anatomy; therefore, the AHA guidelines recommend focusing on the RCA and LAD segments instead [10,27]. In the LAD segment, except for the Kurotobi (30.7%) and Olivieri (29.3%) formulas, aneurysm prevalence was higher (36–38.7%) compared to the JMH criteria (26.7%). For LCx aneurysms, the Dallaire and Kobayashi models demonstrated similar prevalence rates of aneurysms compared to the JMH criteria.

In comparing Z score formulas, more evident variability was observed at higher Z score values, especially for medium and large aneurysms (Z score > 5), as shown in the Bland–Altman plots. This finding is consistent with previous studies from populations with different ethnic backgrounds [24,25,26,28,29]. The increasing disagreement between Z score formulas at higher CA dimensions has important clinical implications. In patients with markedly enlarged CAs, the choice of formula may influence severity classification and, consequently, therapeutic decisions such as antithrombotic management or consideration of interventional or surgical approaches. This variability highlights that, although Z scores provide a valuable standardized framework, they cannot substitute for a comprehensive assessment of CA anatomy. Evaluating CAAs within the context of overall coronary architecture, supported by advanced imaging modalities, remains essential for accurate risk stratification and long-term management.

Pairwise comparisons and agreement analyses revealed significant disparities between specific Z score formulas in the severity-stratified prevalence of CAA. The lowest level of agreement was observed between the Kobayashi and Kurotobi formulas in the LMCA segment. Olivieri and Dallaire’s formulas did not indicate high agreement in any dimensions, suggesting potential limitations in accurately classifying aneurysm severity. There was notable agreement between the McCrindle and Dallaire formulas, particularly in the RCA and LAD segments, although only moderate agreement was found in the LMCA. This pair of formulas graphically showed low variability and narrow limits of agreement in RCA. However, as mentioned, the LMCA segment exhibited a mean difference below zero, indicating a systematic negative bias due to the lower values produced by the McCrindle formula. This could potentially influence not only the diagnosis of KD but also long-term management and treatment decisions, such as the need for anticoagulation in addition to antiplatelet therapy. Given the LMCA’s critical role in supplying blood to large areas of the heart, preventing cardiac events like CA thrombosis is especially important. In contrast, the de Zorzi and Kurotobi formulas demonstrated strong agreement across all dimensions. Importantly, although many published works in this field continue to report results based on de Zorzi formula, it has notable methodological limitations. Particularly, its linear structure and reliance on fixed standard error estimates, which fail to capture the complexity of growth physiology. In addition, this formula is now considered outdated and of limited relevance in contemporary clinical and research practice. Furthermore, the Kobayashi and Dallaire formulas showed similar levels of agreement, but they also included the LCx segment. These formulas provide normative data for the LCx, making them valuable tools for comprehensive assessment [14,18]. Including all major coronary segments in the analysis allows for a complete picture of patient risk, supporting more informed treatment decisions, as the risk of CA events rises with the extent of CA aneurysm involvement [30]. Furthermore, the Bland–Altman plots indicated that the Kobayashi and Dallaire formulas exhibited low variability for both “no aneurysm” and “dilation/small aneurysm” categories, with relatively narrow limits of agreement, particularly for RCA and LMCA.

In contrast to our findings, Kim et al. observed different aneurysm values between the Dallaire and Kobayashi formulas in the LCx segment within a KD population of South Korea [18]. However, in a retrospective study involving 568 American and 514 Japanese patients with KD, Ogata et al. compared the diagnostic efficacy of the Kobayashi and Dallaire formulas and found that both Z score methods performed equally well in patients from the United States and Japan [31]. The AHA defined the two formulas as the most rigorous systems, based on larger populations. With careful statistical modeling, Kobayashi used a lambda-mu-sigma method for regression analysis of BSA and Dallaire using a square root function of BSA [1].

These findings are closely aligned with the recently updated 2024 AHA guidelines, which emphasize the use of Z score–based classification for all pediatric KD patients. The AHA recommends that each center adopt a single, validated Z score model to ensure consistency in clinical practice and research [8]. While multiple formulas are available, none have been universally endorsed, and model selection should consider population-specific validation and local context. Model choice influences not only diagnosis but treatment thresholds for anticoagulation (Z score ≥ 10). In our cohort, up to 15% of borderline LMCA cases were reclassified depending on model choice, potentially altering therapy under AHA guidance.

In this regard, our results reinforce the clinical applicability of the Kobayashi and Dallaire formulas in European settings, particularly among Caucasian children. These models not only demonstrated the highest level of agreement across all major CA segments but also provided stable Z scores with minimal variability, especially in the RCA and LMCA. Still, as pointed out by Lorenzoni and colleagues after comparing four different Z score models (among them Dallaire’s) in KD children from US’s hospitals [29], further long-term outcome research and international consensus efforts are needed to establish a “gold standard” approach for CA normalization in KD children. This would help minimize clinically important variability in diagnosing and managing CA abnormalities. For practical application, the International KD Society (www.ikds.org) has developed a tool that generates user-friendly comparative Z score outputs [32]. This tool incorporates formulas such as those by Kobayashi and Dallaire, as well as a more recent model based on a Canadian reference sample of over 20,000 children, which accounts not only for body size but also for additional confounding variables such as body mass index and sex [33].

This study has some limitations. First, specific data on CA dimensions, along with the corresponding anthropometric measurements required to calculate Z scores, were available for 309 of the 351 KD patients, which may have introduced selection bias. This does not necessarily mean that CA measurements were not performed for the remaining patients, as the attending physician often recorded them simply as “normal CA dimensions”. Such under-reporting of normal findings could bias the results toward an overestimation of pathological coronary dimensions. We strongly encourage pediatric cardiologists to measure CA diameters precisely, even when appearing normal, as accurate dimensions are essential for calculating reliable Z scores and detecting subtle abnormalities that might otherwise be missed. In addition, because CA measurements were more often obtained in patients with suspected coronary involvement, the prevalence of CAA in our cohort is likely overestimated compared with a population-based study. Nevertheless, since this limitation applies equally to all Z score methods, it does not affect the comparative outcomes, which remain the focus of this study. Furthermore, our dataset contains predominantly LMCA measurements, with fewer data available for the LAD and LCx. Given that LMCA is considered less reliable for Z score assessment, this raises a concern that the evaluation of the left CA in our cohort may be less robust. We therefore encourage clinicians to place greater emphasis on LAD, and potentially LCx measurements rather than relying solely on LMCA. Finally, it is also important to note that the echocardiographic data in this study were not obtained in a standardized manner or acquired using a single echocardiography machine. Nonetheless, this study reflects real-world, as it includes echocardiographic data from an active, population-based surveillance study in Germany. Accurate measurement of CA dimensions is increasingly feasible as advancements in echocardiography now provide high-resolution images capable of assessing even CA intima-media thickness, which also appears to be affected in patients with KD [34].

## 5. Conclusions

Accurate diagnosis of CAAs in patients with KD is essential for diagnostic accuracy, timely decisions regarding treatment intensification and long-term care management, as these patients may require additional clinical interventions and cardiology follow-up. In our German KD cohort, we observed notable variability in CAA outcomes depending on whether JMH criteria or different Z score formulas were applied, particularly for medium and large aneurysms. The Kobayashi and Dallaire formulas yielded the most comparable results across major coronary segments, though this does not necessarily confirm their superiority in identifying true aneurysms. Consistent use of a single Z score formula enhances comparability and supports more standardized patient assessment. Larger validation studies and a comprehensive European normative dataset, based on standardized echocardiographic protocols, are needed to optimize Z score application and enhance care for KD patients.

## Figures and Tables

**Table 1:** Demographic and clinical characteristics of the study population (*n* = 309).

	*n*	
Males	309	204 (66)
Age at manifestation (months)	309	32.3 (42.0)
Height (cm)	309	95.0 (31.6)
Weight (kg)	309	13.5 (8.0)
BSA (m^2^)	309	0.601 (0.28)
Proximal RCA (mm)	226	2.45 (1.42)
Proximal LMCA (mm)	252	3.00 (1.28)
Proximal LAD (mm)	75	2.20 (1.42)
Proximal LCx (mm)	36	2.035 (1.50)

Data are expressed as counts (percentage) or median (interquartile range). BSA, body surface area; LAD, left anterior descending; LCx, left circumflex; LMCA, left main coronary artery; RCA, right coronary artery.

**Table 2:** Comparison of RCA, LMCA, LAD and LCx dilation or aneurysm prevalence according to JMH criteria and six Z score formulas.

	Prevalence (%)	Z Score Formulas	Prevalence (%)	*p* Value
**RCA dilatation/aneurysm (n = 226)**				
Japanese Criteria	32.7	Kobayashi	39.8	**<0.001**
		de Zorzi	38.9	**<0.001**
		Kurotobi	37.2	**0.013**
		McCrindle	37.6	**0.013**
		Olivieri	30.5	0.405
		Dallaire	35.0	0.359
**LMCA dilatation/aneurysm (n = 252)**				
Japanese Criteria	47.6	Kobayashi	44.8	0.324
		de Zorzi	29.4	**<0.001**
		Kurotobi	25.8	**<0.001**
		McCrindle	30.6	**<0.001**
		Olivieri	29.4	**<0.001**
		Dallaire	42.9	**0.065**
**LAD dilatation/aneurysm (n = 75)**				
Japanese Criteria	26.7	Kobayashi	36.0	**0.016**
		de Zorzi	37.3	**0.008**
		Kurotobi	30.7	0.250
		McCrindle	38.7	**0.004**
		Olivieri	29.3	0.625
		Dallaire	38.7	**0.004**
**LCx dilatation/aneurysm (n = 36)**				
Japanese Criteria	25.0	Kobayashi	33.3	0.250
		Dallaire	33.3	0.250

Data are expressed as prevalence (%). Statistical differences in aneurysm prevalence were assessed by the paired McNemar’s *t*-test. Significant *p*-value in bold.

**Table 3:** Z score values for RCA, LMCA, LAD and LCx according to different formulas.

	Kobayashi	de Zorzi	Kurotobi	McCrindle	Olivieri	Dallaire
Z score models for proximal RCA (n = 226)	2.6 ± 2.9	2.5 ± 3.1	2.5 ± 2.6	2.8 ± 3.8	1.5 ± 2	2.6 ± 3.4
Z score models for proximal LMCA (n = 252)	2.7 ± 2.7	1.9 ± 2.7	1.8 ± 2	1.9 ± 3	1.7 ± 2.1	2.8 ± 3.2
Z score models for proximal LAD (n = 75)	2.1 ± 2.9	2.7 ± 3.9	2.1 ± 2.9	3.1 ± 5.4	1.6 ± 2.7	3 ± 4.1
Z score models for proximal LCx (n = 36)	1.7 ± 2.9					2.5 ± 4.3

Data are expressed as mean ± standard deviation. LAD, left anterior descending; LCx, left circumflex; LMCA, left main coronary artery; RCA, right coronary artery. *p*-values for all pairwise comparisons are provided in Table S1. The RCA was the segment showing fewer mean differences between the formula pairs.

**Table 4:** Analysis of agreement for the classification of aneurysm severity.

	RCA		LMCA		LAD		LCx	
	Value of Agreement	Level of Agreement	Value of Agreement	Level of Agreement	Value of Agreement	Level of Agreement	Value of Agreement	Level of Agreement
Kobayashi—de Zorzi	0.588	Moderate	0.467	Moderate	0.779	Substantial	-	-
Kobayashi—Kurotobi	0.695	Substantial	0.397	Fair	0.723	Substantial	-	-
Kobayashi—McCrindle	0.754	Substantial	0.542	Moderate	0.649	Substantial	-	-
Kobayashi—Olivieri	0.476	Moderate	0.468	Moderate	0.657	Substantial	-	-
Kobayashi—Dallaire	0.765	Substantial	0.831	Almost perfect	0.745	Substantial	0.857	Almost perfect
de Zorzi—Kurotobi	0.799	Substantial	0.836	Almost perfect	0.722	Substantial	-	-
de Zorzi—McCrindle	0.664	Substantial	0.684	Substantial	0.688	Substantial	-	-
de Zorzi—Olivieri	0.563	Moderate	0.640	Substantial	0.519	Moderate	-	-
de Zorzi—Dallaire	0.659	Substantial	0.486	Moderate	0.741	Substantial	-	-
Kurotobi—McCrindle	0.714	Substantial	0.717	Substantial	0.559	Moderate	-	-
Kurotobi—Olivieri	0.627	Substantial	0.718	Substantial	0.783	Substantial	-	-
Kurotobi—Dallaire	0.731	Substantial	0.402	Moderate	0.576	Moderate	-	-
McCrindle—Olivieri	0.417	Moderate	0.770	Substantial	0.406	Moderate	-	-
McCrindle—Dallaire	0.834	Almost perfect	0.569	Moderate	0.870	Almost perfect	-	-
Olivieri—Dallaire	0.544	Moderate	0.453	Moderate	0.425	Moderate	-	-

Data are expressed as Cohen’s Kappa coefficients. The agreement levels were as followed: poor agreement < 0; slight agreement = 0–0.20; fair agreement = 0.21–0.40; moderate agreement = 0.41–0.60; substantial agreement = 0.61–0.80; almost perfect agreement = 0.81–1. LAD, left anterior descending; LCx, left circumflex; LMCA, left main coronary artery; RCA, right coronary artery.

## Data Availability

The original contributions presented in this study are included in the article/Supplementary Material. Further inquiries can be directed to the corresponding author.

## Supplementary Materials

The following supporting information can be downloaded at: https://www.mdpi.com/article/10.3390/jcm14186581/s1, Table S1: Paired analysis of mean differences in Z score values across the CA segments; Figure S1: Bland–Altman plots comparing five pairs of Z scores formulas across the CA segments (RCA, right coronary artery; LMCA, left main coronary artery; LAD, left anterior descending). For each CA, pairwise differences were computed by subtracting the first author’s values from the second author’s values, specifically: Kobayashi–McCrindle, Kobayashi–Olivieri, de Zorzi–Kurotobi, de Zorzi–McCrindle, de Zorzi–Olivieri. Dotted line represents average difference in Z scores, and solid lines represent limits of agreement for the average difference in Z scores; Figure S2: Bland–Altman plots comparing five pairs of Z scores formulas across the CA segments (RCA, right coronary artery; LMCA, left main coronary artery; LAD, left anterior descending). For each CA, pairwise differences were computed by subtracting the first author’s values from the second author’s values, specifically: de Zorzi–Dallaire, Kurotobi–McCrindle, Olivieri–Dallaire, Kurotobi–Olivieri, Kurotobi–Dallaire. Dotted line represents average difference in Z scores, and solid lines represent limits of agreement for the average difference in Z scores; Figure S3: Bland–Altman plots comparing McCrindle–Olivieri pair of Z scores formulas across the CA segments (RCA, right coronary artery; LMCA, left main coronary artery; LAD, left anterior descending). For each CA, the differences were calculated by subtracting McCrindle’s values from Olivieri’s values. Dotted line represents average difference in Z scores, and solid lines represent limits of agreement for the average difference in Z scores.

## Clinical Trial Registration

German clinical trial registration, DRKS00010071 http://apps.who.int/trialsearch/Trial2.aspx?TrialID=DRKS00010071 (accessed on 4 August 2025).

## Abbreviations

The following abbreviations are used in this manuscript:

AHA	American Heart Association
BSA	body surface area
CAA	coronary artery abnormalities
ESPED	German Pediatric Surveillance Study for Rare Diseases
JMH	Japanese Ministry of Health
KD	Kawasaki disease
LAD	left anterior descending
LCx	left circumflex
LMU	Ludwig Maximilian University
LMCA	left main coronary arteries
RCA	right coronary arteries
SD	standard deviation
