# Supplementary material for: Diagnosis of Coronary Artery Aneurysm in a Caucasian Population Cohort: Evaluating the Agreement Between Japanese Criteria and Different Z Score Formulas
Source: J Clin Med. 2025 Sep 18;14(18):6581. doi: 10.3390/jcm14186581 (PMC12471227; doi:10.3390/jcm14186581)
Supplement: Supplementary file 1 [file jcm-14-06581-s001.zip › jcm-3841657-supplementary.pdf]

Supplemental Table S1. Paired analysis of mean differences in Z scores values across the CA segments.

	Kobayashi	De Zorzi	Kurotobi	McCrindle	Olivieri
**RCA**					
De Zorzi	0.099				
Kurotobi	**0.021**	0.890			
McCrindle	**0.008**	**<0.001**	**<0.001**		
Olivieri	**<0.001**	**<0.001**	**<0.001**	**<0.001**	
Dallaire	0.540	0.125	0.075	**<0.001**	**<0.001**
**LMCA**					
	Kobayashi	De Zorzi	Kurotobi	McCrindle	Olivieri
De Zorzi	**<0.001**				
Kurotobi	**<0.001**	0.293			
McCrindle	**<0.001**	0.227	0.143		
Olivieri	**<0.001**	**0.003**	**<0.001**	**0.001**	
Dallaire	**0.011**	**<0.001**	**<0.001**	**<0.001**	**<0.001**
**LAD**					
	Kobayashi	De Zorzi	Kurotobi	McCrindle	Olivieri
De Zorzi	**<0.001**				
Kurotobi	0.896	**<0.001**			
McCrindle	**0.002**	0.127	**<0.001**		
Olivieri	**<0.001**	**<0.001**	**<0.001**	**<0.001**	
Dallaire	**<0.001**	**0.003**	0.075	0.876	**<0.001**
**LCx**					
Dallaire	**0.011**				

Data are shown as p-value for every pair test. Paired samples t-test was used to assess the Z score mean differences. Significant P-value in bold.

Figure S1

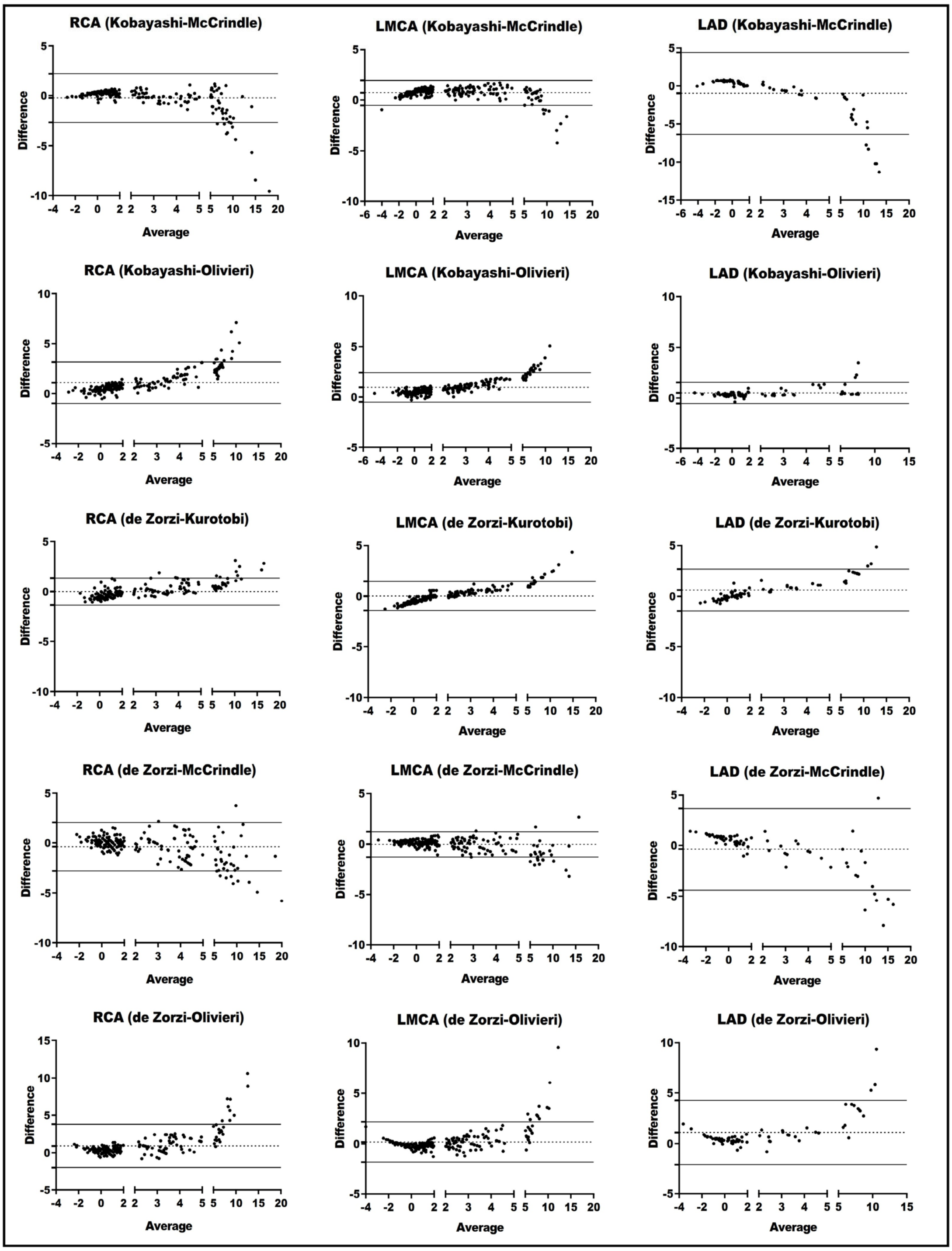

Figure S1: Bland-Altman plots comparing five pairs of Z scores formulas across the CA segments (RCA, right coronary artery; LMCA, left main coronary artery; LAD, left anterior descending). For each CA, pairwise differences were computed by subtracting the first author's values from the second author's values, specifically: Kobayashi-McCrindle, Kobayashi-Olivieri, de Zorzi-Kurotobi, de Zorzi-McCrindle, de Zorzi-Olivieri. Dotted line represents average difference in Z scores, and solid lines represent limits of agreement for the average difference in Z scores.

Figure S2

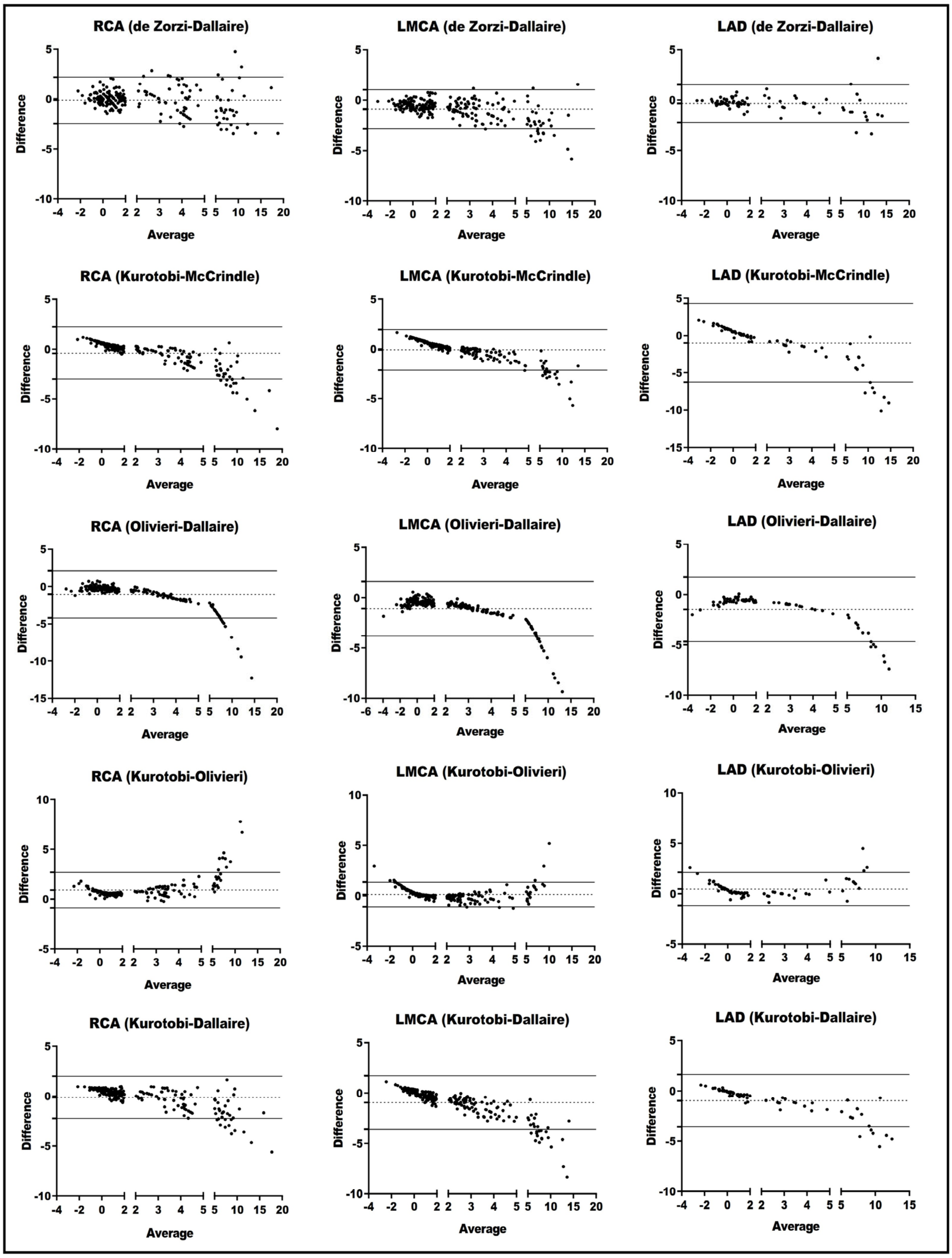

Figure S2: Bland-Altman plots comparing five pairs of Z scores formulas across the CA segments (RCA, right coronary artery; LMCA, left main coronary artery; LAD, left anterior descending). For each CA, pairwise differences were computed by subtracting the first author's values from the second author's values, specifically: de Zorzi-Dallaire, Kurotobi-McCrindle, Olivieri-Dallaire, Kurotobi-Olivieri, Kurotobi-Dallaire. Dotted line represents average difference in Z scores, and solid lines represent limits of agreement for the average difference in Z scores.

Figure S3

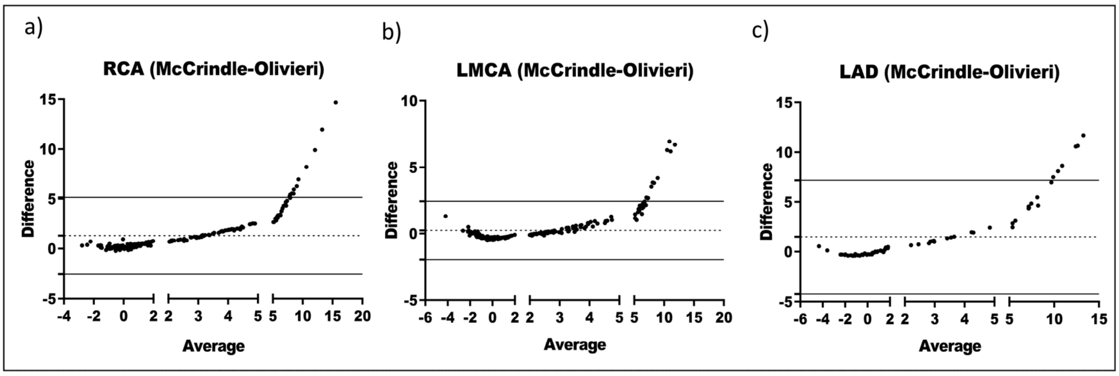

Figure S3: Bland-Altman plots comparing McCrindle-Olivieri pair of Z scores formulas across the CA segments (RCA, right coronary artery; LMCA, left main coronary artery; LAD, left anterior descending). For each CA, the differences were calculated by subtracting McCrindle's values from Olivieri's values. Dotted line represents average difference in Z scores, and solid lines represent limits of agreement for the average difference in Z scores.
